# Mapping vocal interactions in space and time differentiates signal broadcast versus signal exchange in meerkat groups

**DOI:** 10.1098/rstb.2023.0188

**Published:** 2024-06-23

**Authors:** Vlad Demartsev, Baptiste Averly, Lily Johnson-Ulrich, Vivek H. Sridhar, Leonardos Leonardos, Alexander Q. Vining, Mara Thomas, Marta B. Manser, Ariana Strandburg-Peshkin

**Affiliations:** ^1^ Department of Biology, University of Konstanz, Konstanz 78464, Germany; ^2^ Centre for the Advanced Study of Collective Behaviour, University of Konstanz, Konstanz 78464, Germany; ^3^ Department for the Ecology of Animal Societies, Max Planck Institute of Animal Behavior, Konstanz 78467, Germany; ^4^ Kalahari Research Centre, Van Zylsrus 8467, South Africa; ^5^ Department of Evolutionary Biology and Environmental Studies, University of Zurich, Zurich 8057, Switzerland; ^6^ Animal Behavior Graduate Group, University of California, Davis, CA 95616, USA; ^7^ Interdisciplinary Center for the Evolution of Language, University of Zurich, Zurich 8057, Switzerland

**Keywords:** communication, bioacoustics, vocal interactions

## Abstract

Animal vocal communication research traditionally focuses on acoustic and contextual features of calls, yet substantial information is also contained in response selectivity and timing during vocalization events. By examining the spatiotemporal structure of vocal interactions, we can distinguish between ‘broadcast’ and ‘exchange’ signalling modes, with the former potentially serving to transmit signallers’ general state and the latter reflecting more interactive signalling behaviour. Here, we tracked the movements and vocalizations of wild meerkat (*Suricata suricatta*) groups simultaneously using collars to explore this distinction. We found evidence that *close calls* (used for maintaining group cohesion) are given as signal exchanges. They are typically given in temporally structured call–response sequences and are also strongly affected by the social environment, with individuals calling more when they have more neighbours and juveniles responding more to adults than the reverse. In contrast, *short note calls* appear mainly in sequences produced by single individuals and show little dependence on social surroundings, suggesting a broadcast signalling mode. Despite these differences, both call categories show similar clustering in space and time at a group level. Our results highlight how the fine-scale structure of vocal interactions can give important insights into the usage and function of signals in social groups.

This article is part of the theme issue ‘The power of sound: unravelling how acoustic communication shapes group dynamics.'

## 1. Introduction

Understanding the function and potential informational content of animal calls [1,2] requires examining the acoustic structure of signals and the context of their production, but also the temporal and spatial dynamics of signalling events. The interactive flexibility of vocal signalling could serve as an extension of the communication system beyond a fixed signal–function relationship [3]. Thus, examining the temporal and spatial dynamics of vocal exchanges can allow us to gain insight into the function of calls and how they are used to mediate social interactions, beyond the informational content reflected in their acoustic structure.

Signalling events can lie on a spectrum from single-caller signals to multi-participant vocal interactions [4–6], and can range from call-and-response exchanges, through duets with complementary roles for each participant, to highly synchronous vocal choruses [7–9]. Different temporal patterns of signalling events are often driven by different communicational goals, and vary in their potential for exchanging information with specific individuals. Partially or fully overlapping signalling can be beneficial as signals can be perceived from a greater distance [10], but receivers often have a reduced ability to recognize individual signallers [11]. On the other hand, alternating, call-and-response signalling can make it easier for receivers to identify and localize signallers. Such patterns can be beneficial for mate attraction [12] and generally for maintaining contact with designated partners [13].

In a framework similar to the distinction between solo, duet and chorus songs in birds [14] or mammals [15], we can define signalling events according to their organizational properties [10]. We define ‘signal exchanges’ as antiphonal interactions between conspecifics in which each turn mobilizes a co-participant to a ‘response of a particular type’. Under this condition, a signaller is likely to have an expectation of a response [16] and a receiver is stimulated to produce one [17,18] to fulfil the communicational goal of an interaction. Consequently, signal exchanges are likely to demonstrate a consistent temporal structure [13,19], and in many cases, the initiation, response latency, duration and intensity of such interactions may be dependent on the relationship between the participants [18]. In contrast, we can define a divergent pattern, ‘signal broadcasts’, as signalling events that are mainly driven by an environmental stimulus or internal state [20] and are not directly contingent on the perception of conspecifics’ presence or calls. While such events can be intensified by social facilitation [21], there is no response *per se* to preceding signals [6,22] nor are there necessarily expectations for receivers to reply. Independent vocal streams by multiple individuals are therefore expected to result in calling bouts with weak temporal coupling between callers rather than forming distinct interactions with other conspecifics. Examples of signal broadcasts include solo songs and the rare examples of asynchronous choruses [23], as well as other more contextually driven signalling events such as alarm calls [24,25].

Distinguishing between the temporal patterns of signalling behaviour in multi-participant interactions requires constructing an accurate timeline of call emissions from several individuals (ideally, all members of a social unit) to precisely monitor call timing. It is also important to have information about the relative positions of conspecifics during signalling events, as signal relevance is expected to vary between nearby and distant individuals. Finally, pre-existing knowledge on the composition and function of the species vocal repertoire is needed to assess the dynamics of calling events from informational and contextual perspectives.

In this work, we fulfilled the above requirements by collecting simultaneous acoustic and spatial tracking data on groups of meerkats (*Suricata suricatta*) in their natural environment. To do so, we deployed collars on all or most members of meerkat groups that recorded continuous audio data and sampled GPS positions every second. We used this unique dataset to investigate the spatiotemporal structure of multi-participant signalling events for different call types. Investigating the detailed temporal and spatial dynamics of calling allowed us to disentangle signal broadcasts from signal exchanges, while also linking the observed patterns to the proposed function of the emitted signals [26].

Meerkats are social mongooses native to southern Africa. Their complex social system, extensive vocal repertoire and tractability of observation have made them one of the most intensively studied wild mammalian species in the past quarter of a century. Most of meerkats’ behaviour is accompanied by calling, and their vocal repertoire has been well described in terms of acoustic structure, size and contextual function [27–29]. The two most frequently emitted call types in the meerkat vocal repertoire are ‘close calls’ (cc [29–31]) and ‘short note calls’ (sn [32–34]). Adult individuals emit close calls mainly during foraging, at an average rate of 6 calls min^–1^ [35]. Close calls function to maintain group cohesion, with higher individual call rates when meerkats are foraging closer together [36], and these ‘vocal hotspots’ have been shown to guide distant individuals towards them [37]. Additionally, spatially isolated individuals (>10 m to nearest neighbour) showed a tendency to increase their close call rates, perhaps to attract group members and prevent further separation [36].

Short note calls have a broader behavioural context in meerkats. Several sequential variations of short notes are produced in a sentinel (guarding) context and, dependent on the call subtype, function as ‘all-clear’ calming or low-level warning signals [33,38]. Outside of the sentinel context, short notes are emitted during meerkats' morning sunning behaviour and are hypothesized to have a bonding and appeasing function [32]. Additionally, short notes have been observed to be emitted during fast, directed travel bouts [34], which often involve multiple individuals or the entire group.

The aims of this study were twofold: (i) to establish an analytical framework for characterizing different spatiotemporal patterns in multi-participant signalling events; and (ii) to link these spatiotemporal patterns to their function and the communication goals of the signallers. Within the meerkat study system, we expected that close calls, previously reported as contact and separation avoidance calls, would form *signal exchanges*—structured vocal interactions with neighbouring conspecifics that are affected by social surroundings [36,37]. With regard to the short note calls, given the diverse behavioural contexts in which they are produced, we had no defined predictions about short note calling patterns. However, the comparable frequency of close calls and short note calls in the meerkat vocal repertoire allowed for a direct comparison and exploration of differences in their spatiotemporal patterns.

## 2. Methods

### (a) Field procedures

We collected data between June and August in 2017 and 2019 at the Kalahari Research Center, in Kuruman River Reserve, Northern Cape, South Africa. We carried out six collar deployments (3 in 2017 and 3 in 2019) in meerkat groups of 7–18 individuals, chosen by the level of habituation (allowing short-range access, continuous observation and tag deployment via magnetic clasps without capture). All procedures were approved by ethical committees of the University of Pretoria, South Africa (permit: EC031-17) and the Northern Cape Department of Environment and Nature Conservation (permit: FAUNA 1020/2016).

### (b) Tag design

Tracking collars consisted of a GPS unit (Gipsy 5, TechnoSmArt Europe Srl, Italy) with a miniature audio recorder (Edic-mini Tiny+ A77, TS-Market Ltd., Russia), mounted on 5 mm wide leather strap and weather sealed by two-part epoxy glue. We punctured a small hole in the epoxy layer at the location of the microphone to enable sound passage. The audio unit was positioned below the chin of the animal and the GPS unit and antenna were positioned at the back of its head for improved reception. Assembled tags weighed 22–24 g, well below the accepted threshold of 5% body weight of the animal [39]. For juvenile meerkats too small to wear a GPS/audio collar, we instead fitted a lighter (8–10 g) collar containing the audio unit only.

To quantify the relative spatial error of our GPS tags, we measured the distance between pairs of tags when they were placed at the same location (stationary error) and when they were moved together along the same track (moving error) at the field site. The median (+ interquartile range) relative error was 3.4 m (2.0–5.0 m) for stationary tags and 1.0 m (0.7–1.5 m) for moving tags.

### (c) Tag deployment duty cycle and retrieval

Audio recorders were programmed to record at 8 kHz, 16-bit and GPS sample rate was set to 1 fix s^–1^. All units were programmed to activate daily for 3 h (electronic supplementary material, table S1), during times when meerkats engage in group foraging, out of which 1 (middle) h of audio was analysed. For detailed deployment times and dates, see electronic supplementary material, table S1. For detailed collar design and deployment procedures, see Averly *et al.* [40].

Nine animals that could not be collared, including one individual per group that already wore a radio collar for locating the group in the field, were recorded by a human observer in a continuous follow session. The focal-follow audio was recorded using a Marantz PMD-661 solid-state digital recorder (Marantz, Japan) and a directional Sennheiser ME66 microphone with K6 power module (Sennheiser electronic GmbH, Germany), sampling rate 44.1 kHz, 16-bit. The microphone was placed on a telescopic boom pole and held at ~30 cm from the focal animal. A GPS receiver was attached to the end of the microphone, functioning in the same mode as the units in the collar tags.

### (d) Synchronization of audio recordings

To ensure the accurate synchronization of audio files collected by different audio recorders, which could suffer from internal clock drift, we used a ‘talking-clock’ Android app (Talk! Stopwatch & Timer), which itself was synchronized with UTC time using a GPS clock. The app was programmed to produce a ‘ping’ and announce the time through a speaker every 90 s, and the speaker was carried by one of the observers in the group to ensure all meerkat collars recorded the sound. Meerkats are generally habituated to the presence of humans and occasional sounds from electronic devices such as radios, and they showed no visible response to the synchronization pings. Synchronization pings were later retrieved from the recorded audio and their known time stamp (UTC) was used for calculating the corrected time stamp of the recorded meerkat calls. The time stamp correction was done separately for each recorder unit to ensure accurate synchronization despite potentially different rates of clock drift.

### (e) Audio data processing

The recorded audio files were reviewed in Adobe Audition, build 13.0.8.43 (Adobe Inc., San Jose, CA, USA). Spectrograms were generated using a non-overlapping Blackman-Harris windowed samples 0.032 s (256 samples) resulting in spectrograms with a frequency resolution of 31.25 Hz every 0.032 s. Call types were identified and categorized manually by visual–auditory inspection based on a previously established library of meerkat vocal repertoire [34] and labelled using the Adobe Audition markers function. The call identification was done by six trained assistants and verified for consistency by V.D., B.A. or L.L. Subsequent verification of inter-observer consistency was performed. 2.1% of the labelled audio material was randomly selected, anonymized and assigned to a different observer for repeated labelling. The original call classification and the verification results were tested for inter-observer agreement by calculating Cohens’ Kappa coefficient [41]. With a sample of 1504 calls that were labelled by two independent labellers, the calculated Kappa scores indicated substantial agreement between labellers (Kappa = 0.72, *z* = 44.7). Kappa calculations were done in R, v. 4.3.1 [42] (package irr v. 0.84.1 [43]).

### (f) Identifying focal and non-focal callers

Since wearable audio tags often record calls of nearby conspecifics, the identified and labelled vocalizations were marked as focal (call produced by the animal wearing the audio tag) or non-focal (call produced by a near neighbour). The distinction between focal and non-focal calls was based on manual amplitude comparison by a human labeller and on automated comparisons of both spectral and temporal features of the calls. See electronic supplementary methods for further details.

### (g) GPS positioning

To increase the reliability of the GPS data, all fixes taken 60 s before and 60 s after a lost signal instance, or with fewer than five satellites detected, were omitted from the dataset. Likewise, instances of biologically unlikely speeds (more than 10 m between two fixes) were omitted. Coordinates were converted from WGS84 to UTMS34 [44] for convenience of spatial analyses.

### (h) Data analysis

From the time-synched audio streams, we reconstructed a time series of each call produced by each individual, its start and end time (synchronized to UTC time) and its call type (see table 1). As some calls comprised several distinct units (notes) given in quick succession (with less than 0.1 s of silence between units) each such sequence was regarded as a single call in the analysis of call-type transitions and caller sequences. The analyses looking at spatiotemporal dynamics and clustering of calls were performed on the note level. To simplify analysis and reduce the ambiguity of call-type identification, the labelled call types were collapsed into the seven most prevalent categories emitted by adult meerkats. Intermediate calls, which were identified by labellers as hybrids between two call categories (5% of the dataset), were assigned to the call category that was more abundantly represented in the meerkat repertoire. Calls that could not be reliably assigned to any of the categories were marked as unknown. Overall, the dataset used here includes a total of 71 043 calls over the course of 26 h of full-group tracking data (electronic supplementary material, table S1).

**Table 1.** Call-type categories used, their abbreviation codes, proposed function and sample sizes in the dataset.

call category	call code	function/emission context	comprising call types	number of identified calls
close call	cc	group cohesion, foraging [36,37]	cc and hybrid cc	47 083
short note	sn	appeasing, sentinel, sunning [32,33]	sn and hybrid sn	15 048
social call	soc	begging, general social interaction [34,45]	soc and hybrid soc	3143
aggression call	agg	physical conflict [34]	agg and chat	1807
move call	mo	movement speed, sentinel [46]	mo and hybrid mo	1650
alarm	al	predator alarm [33,47]	aerial and terrestrial al	1605
lead call	ld	movement initiation [34,48]	ld and hybrid ld	459
unknown	ukn		unidentified calls	248

### (i) Call category transitions in vocal sequences

As an initial step, we first established whether sequentially emitted calls form stable combinations and which call categories in the meerkat vocal repertoire are frequently emitted together. To do so, we examined the transition probabilities between the seven call categories defined for this study. To determine the relevant time frame of call transitions in meerkats we generated a distribution of all inter-call interval times. In 90% of cases, for any given call the next call was produced within 2.31 s ((median = 0.656 s; IQR =1 s)), so as a precaution towards excluding temporally independent signalling events, the upper 10% of the call transition times (>2.31 s) were omitted from subsequent analysis. To examine the occurrence of different call-category pairs we adopted the multiple distinctive collocation analysis (MDCA) described in Bosshard *et al.* [49] and used it for quantifying ‘collocations’ (grammatical constructions) in human [50] and animal [51] communication. Briefly, the method calculates binomial probabilities for every possible call category combination (accounting for sample size) and log-transforms them for estimation of under- or over-representative combinations (the greater the value, the stronger the association between call categories in the combination). As described in the literature, the method assesses the association between sequential call pairs produced by one individual. Here, we adapted the method to also assess the association strength between call pairs when produced by two different callers and repeated the MDCA analysis with two subsets of the data: (i) both calls in the pair were produced by the same individual (self-reply) and (ii) the calls in the pair were produced by different individuals (non-self-reply).

### (j) Caller transitions in vocal sequences

**Figure 1.**
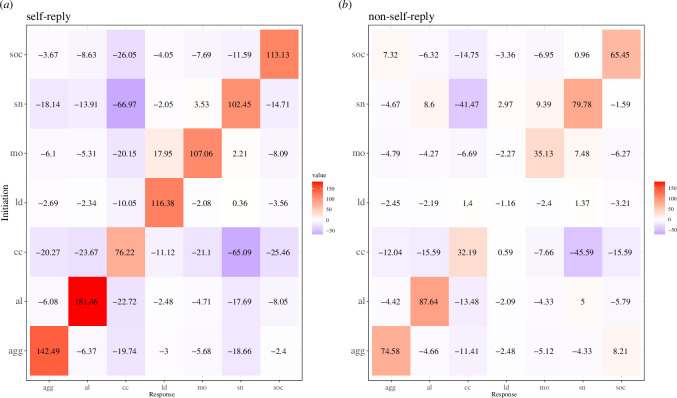
Transition matrices for (***a***) self-reply call pairs (in which two sequential calls were emitted by the same individual) and (***b***) non-self-reply call pairs (in which sequential calls were emitted by different individuals). The values in the tiles represent the relative association between initiation call type (***x*** *)* and the response call type (***y***) while accounting for the total number of transitions with initiation call type (***x*** *)*. The colours denote over- (orange) or under- (purple) representation of each call pair combination. The values correspond to *p* estimation significance levels (i.e. pbin > 3: *p* < 0.001; > 2: *p* < 0.01; > 1.3: *p* < 0.05; if < 1.3: NS). The script was adapted from Gries [52].

Calls produced as part of signal exchanges should show evidence of caller transition (i.e. individuals calling after others have called) within a relatively short time period. To assess whether close calls (cc) are likely to appear in structured call-and-response signal exchanges, and more generally, how commonly caller transitions occur in meerkats across different call categories, we calculated the proportions of self-reply and non-self-reply call pairs in our data. Given the strong tendency for calls to be followed by calls of the same category in the meerkat repertoire (figure 1), we assessed the rate of self-reply versus replies to others (non-self-reply) for homogeneous call pairs representing the five call categories that appeared most frequently in our dataset (cc–cc: 34 428 pairs; sn–sn: 7213 pairs; soc–soc: 1112 pairs; agg–agg: 669 pairs; and al–al: 739 pairs), thus allowing robust inference. Move (mo) and Lead (ld) calls were excluded due to the low sample sizes of homogeneous call pairs for these categories (284 and 51 occurrences, respectively). We calculated the probability of caller transition between consecutive calls of the same category as the proportion of non-self-reply events across all consecutive call pairs of that category. To test for differences between the calculated non-self-reply proportions across call types, we performed a Pearson’s chi-squared test and a post hoc pairwise comparison with Bonferroni correction for multiple testing.

To further investigate the caller transition dynamics of sequential signalling events beyond call pairs, we focussed only on the two most abundant call categories (close calls and short notes), which together represented 87% of the calls in our dataset. The other categories were omitted due to their relative scarcity in our data (table 1). Keeping the maximal inter-call interval at 2.31 s, we extracted all sequences of 2–3 calls of the same category where all callers were within a 10 m radius of the initial caller. This distance threshold was chosen based on the maximal range at which calls were likely to receive responses (see §k). Within these sequences, we then recorded the identities of callers. The first caller in a sequence was designated caller A. The next caller was designated as B (if different from the first caller) and the third caller was designated as caller C (if different from both first and second callers). We also included sequences where no third call was produced within the maximal inter-call interval (denoted here as ‘stop’). Based on these sequences, we calculated the relative proportions for the possible caller combinations of 1–3 callers (A–A–stop, A–A–A, A–A–B, A–B–stop, A–B–A, A–B–B and A–B–C). To assess whether the proportions derived from the data are different from randomly produced calls, we compared the resulting proportions to a null distribution of caller sequences based on permuting the times of calls. For details see electronic supplementary material, figure S2.

### (k) Spatiotemporal dynamics of vocal exchanges

Calls produced as part of a signal exchange are likely to be temporally structured, and potentially more distance-dependent, in comparison to broadcast signals. To investigate the temporal and spatial properties of dyadic call interactions, we measured the rate at which individuals produced calls at different times both leading up to and following calls from their conspecifics, when located at different distances away from them. To estimate typical call–response patterns as a function of the spatial separation, we aggregated data from all pairs of individuals and all calls of the relevant category. First, we identified all instances in which an individual (which we designate as the *caller*) gave a call (the *focal call*) and created time series (with *t* = 0 as the time when the focal call was given) that included the call rates of other nearby individuals (designated as potential responders). Potential responder call rates were binned based on the distance to the caller (0–2, 2–5, 5–10 and 10–50 m), and we used a kernel-based method to quantify the rate of calling of potential responders both before and after the focal call. Because we lacked GPS data for juvenile meerkats, this analysis only included calls produced by adults and subadults large enough to wear a collar.

In a similar analysis to the one described above, we also investigated whether call–response patterns in each time series differed depending on the age (adult versus juvenile) of both the caller and the potential responder. In this case, we did not subset the data based on distance between caller and responder, since distance information was not available for juvenile meerkats.

For further details on the analytical approach, see electronic supplementary material, figure S1.

### (l) Clustering of calls in space and time

The structure of vocal exchanges can have consequences not only for the individuals directly involved in the exchange, but also for the overall acoustic landscape experienced by group members. This landscape may in turn shape group-level outcomes such as cohesion maintenance and movement decisions [37]. In addition to characterizing the spatiotemporal dynamics of call exchanges at the individual-to-individual level, we therefore also quantified the overall clustering of calls in space and time at an aggregate level. To do so, we first calculated, for every pair of calls in the dataset of a given category (close call or short note), the distance between the caller and the responder at the time of the first call, as well as the time delay between the calls. This analysis therefore excluded any calls for which GPS data were not available (for example, juvenile calls). We then counted the number of pairs of calls that were observed within a given range of distances apart and time delays (i.e. a spatiotemporal bin). We then normalized the number of pairs within each spatiotemporal bin by the total number of pairs of calls in the same time delay range across all distances. This normalized value, which we refer to as *K*, can be interpreted as the extent to which calls are spatially clustered over a given spatial scale, at a given time delay.

Because meerkats form a moving group that is also, in itself, clustered in space and time, we wanted to compare this value to a null expectation of the level of clustering expected if there were no vocal interactions. To generate this null expectation, we created artificially shuffled datasets in which the audio data from each meerkat were randomly combined with the GPS data from another. This permutation preserves the spatial distribution of meerkats and the individual calling sequences, but breaks the link between calling behaviour and spatial configuration. Using these permuted datasets (*n* = 100 permutations), we recalculated the clustering metric *K* at each spatiotemporal bin as described above, then calculated the mean value of *K* for each bin across all permutations. Finally, we took the log ratio of the real data and the permuted data for each spatiotemporal bin. Positive values of the log ratio represent increased clustering relative to the null over a given spatiotemporal scale, whereas negative values represent decreased clustering relative to the null. This analysis thus gives us insight into whether, and over what spatial scales, ‘hotspots’ of calls emerge and how long these hotspots persist [37].

### (m) Call rate as a function of the movement speed and number of nearby neighbours

To give further insight into the social context in which calls were given, we investigated for both close calls and short note calls how the mean call rate varied with individual speed and the number of nearby conspecifics. If calls are given as part of a signal exchange, the rate of calling would be predicted to show dependence on the number of nearby neighbours. Moreover, with increasing speed, the rapidly changing social surroundings might require more frequent updates on the identity and the location of neighbours, thus driving up contact call rates.

For each individual, we first computed its close call rate and short note call rate over time, using a 10 s rolling window. To enable comparisons between individuals with different baseline call rates, we standardized the call rates for both call types by calculating *z* scores for each 10 s window using the formula $\frac{x-\mu }{\sigma }$ , with *x* being the raw call rate in a given window, *µ* being the mean call rate across all time windows and *σ* being the standard deviation. We next computed each focal individual’s speed by taking its displacement over the same 10 s window and dividing by the time elapsed, as well as the number of individuals within 5 m of the focal individual. To reduce bias from any missing data or the combination of multiple contexts, we only included times when at least five individuals in the group were tracked. Also, since meerkats only emit close calls in the foraging context we only included times when at least half of the group members were foraging (had given a close call in the past minute). We also excluded any data from times when alarm calls had been heard from any member of the group in the past minute.

After subsetting to relevant contexts and normalizing call rates as described above, we then assessed the relationship between call rates, speed and nearby neighbours for both call types. For each speed bin (divided into 10 bins based on quantiles of the speed distribution), we computed the mean normalized close call and short note rates across all instances in which a speed in that range was observed. Similarly, we also computed mean normalized call rates as a function of the number of nearby neighbours (bins = 0, 1, 2, 3 and 4 or more neighbours).

To assess whether patterns were consistent across individuals, we computed these means both within each individual and across all individuals. Individuals for which less than 2 h of data were available were excluded from the individual-level computations (leaving *n* = 27 individuals included in this analysis).

All data processing, analysis and plotting was performed in R v. 4.3.1 [42].

## 3. Results

The dataset used in the current analysis includes audio recordings from 38 individual animals (21 males and 17 females) from three different meerkat groups, collected over 26 days (1 h/d). This dataset consists of 71 043 calls that were assigned to seven call categories or annotated as unknown (table 1).

### (a) Call-type transitions in vocal sequences

The overall combinatorial structure of meerkat vocal repertoire represented by the probabilities of sequential emission of various call types demonstrates that homogeneous (same type) call pairs are strongly over-represented for most call types, and in both self-reply and non-self-reply sequences (figure 1).

Move (mo) calls are a noticeable exception in the self-reply sequences. Individuals frequently produced move–lead (mo–ld) call combinations, however, the reverse call order (lead–move) appeared only rarely. In the non-self-reply dataset, lead calls are not immediately followed by calls of other individuals, short note (sn) calls are likely to both precede and follow alarm (al), lead (ld) and move (mo) calls above random chance, but the overall number of these events is relatively low (3.5% from the total call-pair data).

### (b) Caller transitions in vocal sequences

**Figure 2.**
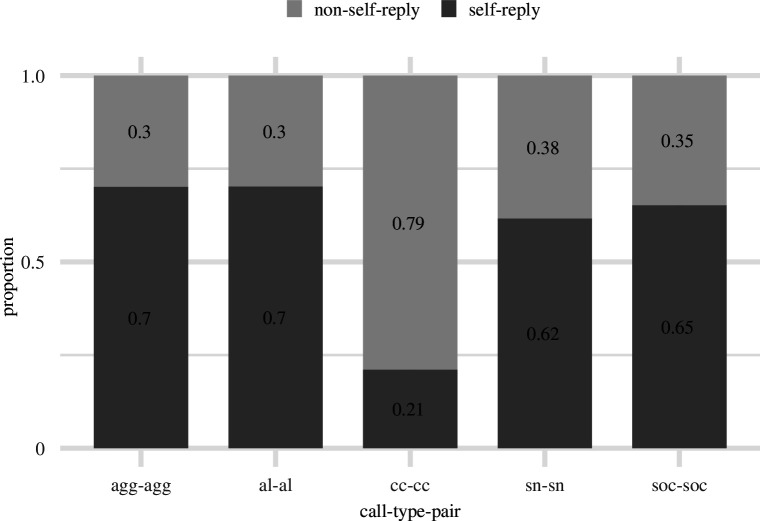
Proportions (*y*-axis) of self-reply (dark grey) and non-self-reply (light grey) call pairs for the five most abundant call types in meerkat vocal repertoire (agg, aggression; al, alarm; cc, close call; sn, short note; soc, social). The values in the stacked bars indicate fractions of the respective condition.

Analysis of caller transitions across the five most common call types showed that close call pairs (cc–cc) demonstrate a caller exchange pattern, with 80% of sequential call pairs showing the participation of two individuals (figure 2). The other four call types show predominantly self-reply transitions, i.e. cases in which two consecutive calls are produced by the same individual. The chi-squared test showed significant between call type differences in self-reply/non-self-reply proportions*: χ* ^2^ = 6432.2, d.f. = 4, *p*‐value < 0.001. A pairwise comparison showed that cc–cc caller transition proportion is significantly different from all other tested call types (sn–sn, al–al, agg–agg and soc–soc), with all pairwise corrected *p* values < 0.001 (electronic supplementary material, table S2).

**Figure 3.**
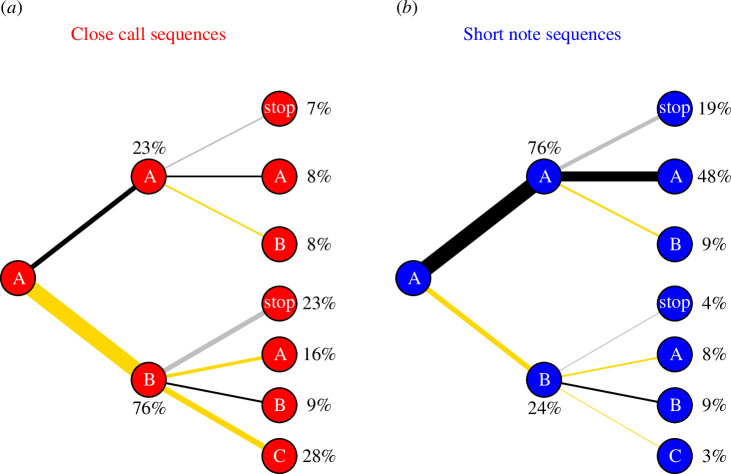
Caller sequences for (***a***) close calls (cc) and (***b***) short note (sn) calls. Same letters denote the same caller identity. Numbers show the proportion of the respective caller sequences (at the end of the respective edge) found in the dataset. Edge thickness indicates the proportion of respective caller sequence. Edges terminating with a ‘stop’ node indicate sequences in which third call was not emitted within the set time and distance limits (see §2). Yellow edges indicate caller transitions.

Expanding the dyadic caller transition analysis to longer sequences of calls shows that close calls (cc) frequently involved caller exchange between two or three individuals (A–B–C, A–B–stop and A–B–A patterns, figure 3*a*) but with only a small proportion of sequences being produced as repetitions by the same caller (A–A–A and A–A–stop patterns). The overall proportion of cc call sequences that involved caller transition (A–B–A, A–B–stop and A–B–C) was significantly over-represented in comparison to the random expectation (*p* = 0.002, electronic supplementary material, figure S2*a*). Short note calls (sn), in contrast, were often emitted by the same caller as sequences of two or three calls (A–A–stop and A–A–A patterns) in a row, and caller exchanges were strongly under-represented (A–B–C, A–B–stop and A–B–A patterns, figure 3*b*, electronic supplementary material, figure S2*b*), supporting the notion of weak temporal contingence on the calls of other conspecifics.

### (c) Spatiotemporal dynamics of responses to conspecific calls

**Figure 4.**
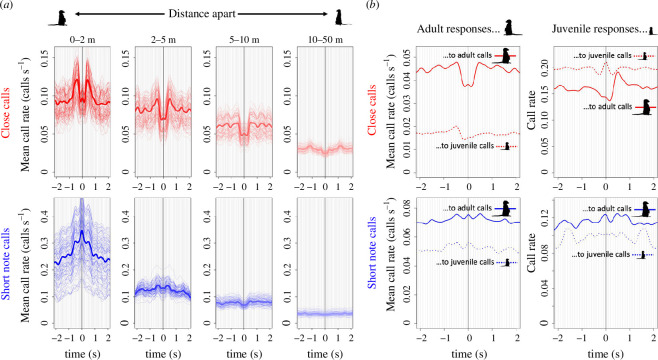
Call–response dynamics in space and time (***a***) and as a function of the individual age (***b***) for close calls (top row, red) and short note calls (bottom row, blue). (***a***) Mean call rate of potential responders relative to a call by a focal caller, as a function of the distance between caller and potential responder (panels) and time relative to the focal call (*x*-axis). Thick lines show mean call rates (per second) taken across all groups and all days. Thin lines show bootstrapped replicates where days were randomly sampled with replacement, taking the same number of days per group as in the full dataset. (***b***) Mean call rate of potential responders relative to a focal call, as a function of the age class of both focal caller and potential responder.

Comparison of the spatial and temporal properties of individual vocal responses to conspecific close calls supports the idea that these calls are emitted as part of a temporally structured call-and-response pattern (figure 4*a*, top). Conspecifics within 0–5 m of a focal caller showed an increased call rate at a characteristic time lag of approximately 400 ms, resulting in symmetrical peaks in close call rate before and after the focal call. The symmetrical pattern results from our inclusion of all possible combinations of callers and responders in our analysis. In other words, the peak after the focal call (approximately +400 ms) demonstrates signalling events where the conspecific called after the focal caller, whereas the peak before (approximately −400 ms) demonstrates events where the conspecific called before the focal caller.

In contrast to close calls, short note calling events demonstrate a different pattern, with a central peak in call rate at time 0. This pattern indicates simultaneous calling behaviour by both focal individuals and their neighbours without a clear call-and-response temporal structure, potentially stimulated by a general environmental or social context (figure 4*a*, bottom). The spatial relevance of both close calls and short note calls is similar, as indicated by reduction in the call rates of neighbours beyond the 5–10 m range (i.e. vocal hotspots) and the flattening of the curves (i.e reduced interaction, see electronic supplementary material, figure S1).

The age class of individuals taking part in the close call interactions had an effect on the emerging call-and-response patterns (figure 4*b*, top), with juveniles more likely to respond to adults than the reverse (time of peak >0). In contrast, there were no clear age-related temporal patterns for short note calls (figure 4*b*, bottom). Across both types of calls, juveniles also called at an overall higher rate than adults (figure 4*b*; note *y*-axis limits).

### (d) Clustering of calls in space and time

**Figure 5.**
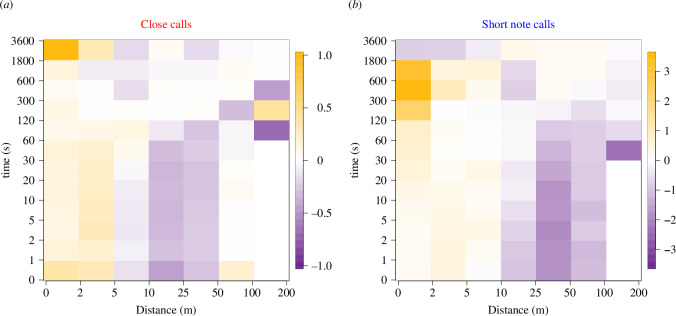
Comparison of call clustering to null expectations based on meerkat positions for close calls (*a*) and short note calls (*b*). Colour represents the log ratio of the observed level of clustering to the expected level of clustering at a given distance (*x*-axis) and temporal (*y*-axis) scale. Orange regions indicate spatial and temporal scales for which pairs of calls are found more often than expected, purple regions show where they are found less often than expected, and white regions show where they are found as often as expected, relative to the distribution of meerkats. Note that all call pairs are considered in the analysis, not solely consecutively produced calls, in order to measure the persistence of call clustering at an aggregate level. Overall, both types of calls tend to be clustered at short spatial scales (up to ~5–10 m). The temporal persistence of the clustering of calls is relatively long, lasting at least 30 min. Note the different colour scales for the two panels, indicating overall greater effects (relative to null expectations) for short note calls than for close calls.

At an aggregate level, close calls and short note calls showed similar spatiotemporal clustering patterns (figure 5). Both types of calls were locally clustered compared to null expectations at a distance of up to 5–10 m, and these clusters persisted over relatively long (>30 min) timescales.

### (e) Call rate as a function of the movement speed and number of nearby neighbours

**Figure 6.**
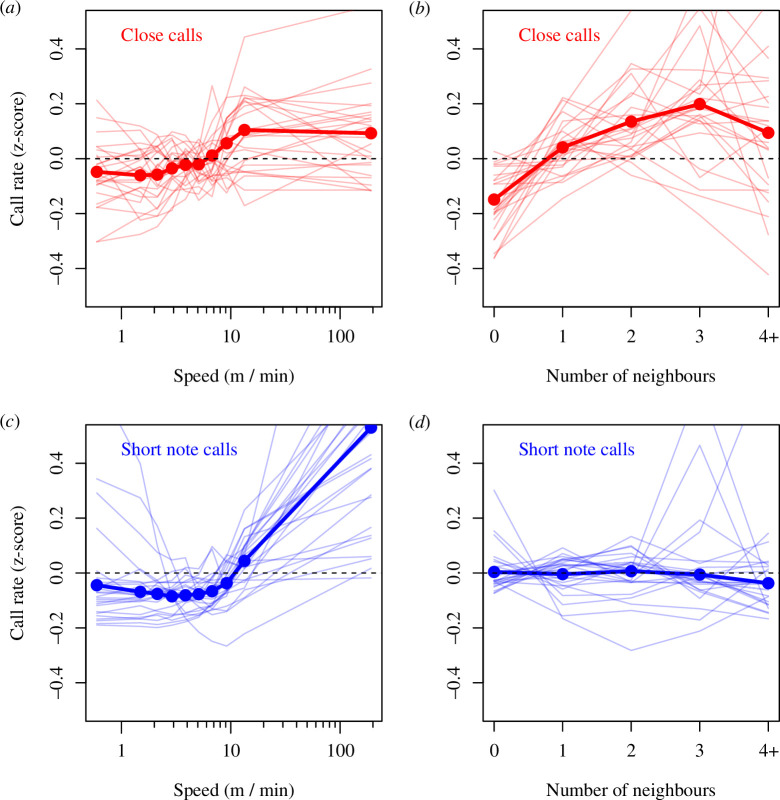
Relationship between call rate and individual speed and number of nearby neighbours (<5 m) differs between close calls and short note calls. Plots show the mean call rate for each individual (thin lines) and across all individuals (thick lines and points) as a function of the individual speed (*a*,*c*: note *x*-axis log scale) and number of tracked neighbours within a 5 m radius (***b,d***), for both close calls (***a,b***) and short note calls (***c,d***). For speeds, data were divided into 10 equally sized bins based on quantiles of the overall speed distribution across all individuals; points are plotted at the middle of each bin. For number of nearby neighbours, data are shown for 0 (43.1% of instances), 1 (29.5%), 2 (15.2%), 3 (7.3%) and 4 or more (4.9%) tracked neighbours.

Individual close call and short note call rates showed a strong dependence on movement speed, albeit on different scales. Close call rates increased at medium movement speeds (below ~10 m/min) but plateaued and potentially decreased during faster movements (figure 6*a*). Short note call rate, in contrast, remained below the baseline during slow and medium movement speeds but dramatically increased for fast movements (above ~10 m/min, figure 6*b*). The number of conspecific neighbours within a 5 m radius had an overall positive effect on individual close call rate, with a gradual increase as a function of the number of neighbours (figure 6*c*). Short note call rate, on the other hand, showed little variation as a function of the number of neighbours (figure 6*d*).

## 4. Discussion

Examining the structure of signalling events in meerkats shows that the two most common call categories, close calls and short note calls, follow different temporal patterns. Close calls, which function to maintain group cohesion [37], seem to be emitted as part of interactive ‘call-and-response’ *signal exchanges*. They show a high probability of caller exchange, with symmetrical peaks indicating temporally structured call–response patterns as well as dependence on the presence and the age class of nearby conspecifics. In contrast, short notes seem to be emitted as part of context-driven *signal broadcast* events. Short note call rates are independent of the number of nearby conspecifics, are mainly produced in sequences by single individuals and do not show temporal structuring typical of call–response patterns. Despite these differences, both call types show similar spatial response ranges. On the collective level, this results in the formation of vocal hotspots for both types of calls—clusters of vocalizations emerging on a local scale (5–10 m) and persisting for up to 30 min.

Signal exchange and signal broadcast offer different potential for identifying and localizing neighbours, and may therefore be linked to different communicational goals and signal functions. Characterizing the temporal structure of signalling events can thus give additional insights into call function and usage beyond what can be gained through analysing acoustic structure and behavioural context.

In the case of meerkat close calls, previous studies have shown that they are individually distinctive [53] and socially facilitated, and that they function to maintain group cohesion and avoid separation [36,37]. However, beyond mediating the risk of separation [36,37], a signal exchange pattern offers the possibility for more detailed mapping of the social surroundings. By reducing signal overlap and mobilizing receiver responses, a signal exchange pattern can facilitate individual identification and localization [11,12]. Such social mapping could be valuable for mediating proximity with particular conspecifics. Strongly bonded individuals might prefer to stay together [54] or, conversely, individuals might avoid proximity to certain group members [55]. Frequently mapping one’s social surroundings could be especially crucial during foraging. For example, contact calls of pied babblers (*Turdoides bicolor*) [55] and green woodhoopoe (*Phoeniculus purpureus*) [56] have been shown to increase spacing during foraging, possibly minimizing food competition. As meerkats normally do not share food and dominants have been observed displacing subordinates from high-value foraging holes [57], a similar competition avoidance function of close calls might be expected. Previous findings do not support close calls as a general mechanism for repelling neighbours in meerkats because they do not avoid proximity with individuals producing contact calls [36]. Nevertheless, meerkats have been shown to respond differently to the close calls of dominant females under conditions of heightened risk of aggression [29], indicating the potential for more selective social monitoring.

The rate at which information on social surroundings needs to be updated should vary as a function of the complexity of the social landscape [58] and how frequently it changes. Having more, constantly moving near-neighbours inevitably increases an individual’s uncertainty about its immediate social surroundings. Further, rapid individual movement increases the rate at which social surroundings are potentially changing. A higher rate of contact call exchange [53,59], as shown here in meerkats, could help overcome these challenges by providing more frequent updates on the positions and identities of the neighbours.

Contact calls, while predominantly allowing individual recognition [59], do not necessarily exhibit signal exchange characteristics [60] as the reunification function can be achieved by other means. For example, chacma baboon (*Papio ursinus*) females respond to infant separation calls by retrieving the vocalizing young rather than by responding vocally [60]. Nevertheless, the same type of signal can demonstrate different temporal patterns depending on the spatial scale considered, with potential functional implications. In meerkats, close calls are structured as signal exchanges at a small scale, perhaps driven by the need for periodic updates on the presence and identity of neighbours [29,61]. At a large scale, the temporal structure becomes less pronounced. At this scale, close calls have been shown to function as an indicator of the group’s core, allowing individual animals to track and follow the group's movement path [37]. As the primary motivation of isolated individuals is to re-join the group, detailed social mapping may be less relevant.

Unlike close calls, short note calling events do not show evidence of structured signalling exchanges, there are no clear sender–receiver pairs and individuals do not seem to demonstrate consistent changes in their calling patterns due to their social environment. As such they are more likely to be a broadcast signal transmitting a signaller’s response to an external event or an indication of its behavioural state, addressed broadly to any receiver.

The sharp increase in short note emission at high movement speeds is a potential marker for a shift in individuals’ behavioural state from foraging to running. Vocalizations commonly accompany transitions between movement states in social animals [62,63], advertising motivation and trajectory of movement [18] and aiding in group navigation during fast travel (e.g. nocturnal flights of bird flocks [64]). While the function of short note emission during movement in meerkats has not been investigated so far, it is possible that these calls might serve as a mechanism for maintaining group cohesion while moving quickly. A high call rate might then arise because individuals need to transmit more frequent orientation markers when changing position rapidly than when moving at a slow pace. The lack of signal exchange patterns in short note sequences could indicate a lower requirement for detailed social monitoring, since unlike during foraging, the potential for competition while on the move is low and coordination of travel is likely to be the main driver behind the signalling event. Alternatively, fast movement might introduce aerobic constraints on vocal production because gait and breathing cycles could be phase locked [65]. As call rate becomes coordinated with stride cycles [66,67] animals might have limited capacity for adjusting their call timing to the calls of conspecifics and maintaining call-and-response patterns.

Whereas our results support that meerkats produce short notes as broadcast signals during rapid movement, an earlier study demonstrated that the same calls emitted while sunning at the burrow show overlap avoidance and a signal exchange pattern [32]. This contrast highlights how characterizing the structure of multi-participant vocal interactions can give insight into the multi-faceted nature of vocal signals. The same signal in different contexts can show different temporal patterns, likely corresponding with shifts in communicational goals and functions.

## Data Availability

The data and scripts used for generating the figures are available at [68]. Electronic supplementary material is available online [69].

## References

[1] Morton ES . 1977 On the occurrence and significance of motivation-structural rules in some bird and mammal sounds. Am. Nat **111** , 855–869. (10.1086/283219)

[2] Briefer EF . 2012 Vocal expression of emotions in mammals: mechanisms of production and evidence. J. Zool **288** , 1–20. (10.1111/j.1469-7998.2012.00920.x)

[3] Clay Z , Archbold J , Zuberbühler K . 2015 Functional flexibility in wild bonobo vocal behaviour. PeerJ **3** , e1124. (10.7717/peerj.1124) 26290789 PMC4540007

[4] Koren L , Geffen E . 2009 Complex call in male rock hyrax (Procavia capensis): a multi-information distributing channel. Behav. Ecol. Sociobiol. **63** , 581–590. (10.1007/s00265-008-0693-2)

[5] Clarke E , Reichard UH , Zuberbühler K . 2006 The syntax and meaning of wild gibbon songs. PLoS One **1** , e73. (10.1371/journal.pone.0000073) 17183705 PMC1762393

[6] Greenfield MD , Tourtellot MK , Snedden WA . 1997 Precedence effects and the evolution of chorusing. Proc. R. Soc. B **264** , 1355–1361. (10.1098/rspb.1997.0188)

[7] Demartsev V , Ilany A , Barocas A , Bar Ziv E , Schnitzer I , Koren L , Geffen E . 2016 A mixed strategy of counter-singing behavior in male rock hyrax vocal competitions. Behav. Ecol. Sociobiol. **70** , 2185–2193. (10.1007/s00265-016-2222-z)

[8] Maynard DF , Ward KAA , Doucet SM , Mennill DJ . 2012 Calling in an acoustically competitive environment: duetting male long-tailed manakins avoid overlapping neighbours but not playback-simulated rivals. Anim. Behav **84** , 563–573. (10.1016/j.anbehav.2012.06.008)

[9] Behr O , Knörnschild M , von Helversen O . 2009 Territorial counter-singing in male sac-winged bats (Saccopteryx bilineata): low-frequency songs trigger a stronger response. Behav. Ecol. Sociobiol. **63** , 433–442. (10.1007/s00265-008-0677-2)

[10] Langmore NE . 2002 Vocal duetting: definitions, discoveries and directions. Trends Ecol. Evol. **17** , 451–452. (10.1016/S0169-5347(02)02611-3)

[11] Wollerman L . 1999 Acoustic interference limits call detection in a Neotropical frog Hyla ebraccata. Anim. Behav. **57** , 529–536. (10.1006/anbe.1998.1013) 10196042

[12] Greenfield MD . 1994 Synchronous and alternating choruses in insects and anurans: common mechanisms and diverse functions. Am. Zool. **34** , 605–615. (10.1093/icb/34.6.605)

[13] Pika S , Wilkinson R , Kendrick KH , Vernes SC . 2018 Taking turns: bridging the gap between human and animal communication. Proc. R. Soc. B **285** , 20180598. (10.1098/rspb.2018.0598) PMC6015850 29875303

[14] Bradley DW , Mennill DJ . 2009 Solos, duets and choruses: vocal behaviour of the Rufous-naped Wren (Campylorhynchus rufinucha), a cooperatively breeding neotropical songbird. J. Ornithol. **150** , 743–753. (10.1007/s10336-009-0393-3)

[15] De Gregorio C , Carugati F , Valente D , Raimondi T , Torti V , Miaretsoa L , Gamba M , Giacoma C . 2022 Notes on a tree: reframing the relevance of primate choruses, duets, and solo songs. Ethol. Ecol. Evol. **34** , 205–219. (10.1080/03949370.2021.2015451)

[16] Cartmill EA , Byrne RW . 2007 Orangutans modify their gestural signaling according to their audience’s comprehension. Curr. Biol. **17** , 1345–1348. (10.1016/j.cub.2007.06.069) 17683939

[17] Stivers T , Rossano F . 2010 Mobilizing response. Res. Lang. Soc. Interact. **43** , 3–31. (10.1080/08351810903471258)

[18] Teixeira da Cunha RG , Byrne RW . 2009 The use of vocal communication in keeping the spatial cohesion of groups: Intentionality and specific functions. In South American primates. Comparative Perspectives in the Study of Behavior, Ecology, and Conservation (eds PA Garber A Estrada , JC Bicca-Marques , EW Heymann , KB Strier ), pp. 341–363. New York, NY: Springer. (10.1007/978-0-387-78705-3)

[19] Todt D , Naguib M . 2000 Vocal interactions in birds: the use of song as a model in communication. In Advances in the study of behavior (eds PJB Slater JS Rosenblatt , CT Snowdon , TJ Roper ), pp. 247–296. Amsterdam, The Netherlands: Elsevier.

[20] Demartsev V , Kershenbaum A , Ilany A , Barocas A , Bar Ziv E , Koren L , Geffen E . 2014 Male hyraxes increase song complexity and duration in the presence of alert individuals. Behav. Ecol. **25** , 1451–1458. (10.1093/beheco/aru155)

[21] Clayton DA . 1978 Socially facilitated behavior. Q. Rev. Biol. **53** , 373–392. (10.1086/410789)

[22] Yoshida S , Okanoya K . 2005 Animal cognition evolution of turn-taking: a bio-cognitive perspective. (10.11225/JCSS.12.153)

[23] Ravignani A , Verga L , Greenfield MD . 2019 Interactive rhythms across species: the evolutionary biology of animal chorusing and turn-taking. Ann. N. Y. Acad. Sci. **1453** , 12–21. (10.1111/nyas.14230) 31515817 PMC6790674

[24] Wilson DR , Evans CS . 2012 Fowl communicate the size, speed and proximity of avian stimuli through graded structure in referential alarm calls. Anim. Behav. **83** , 535–544. (10.1016/j.anbehav.2011.11.033)

[25] Zuberbühler K , Noë R , Seyfarth RM . 1997 Diana monkey long-distance calls: messages for conspecifics and predators. Anim. Behav. **53** , 589–604. (10.1006/anbe.1996.0334)

[26] Demartsev V , Gersick AS , Jensen FH , Thomas M , Roch MA , Manser MB , Strandburg‐Peshkin A . 2023 Signalling in groups: new tools for the integration of animal communication and collective movement. Methods Ecol. Evol **14** , 1852–1863. (10.1111/2041-210X.13939)

[27] Clutton-Brock T , Manser M . 2016 Meerkats: cooperative breeding in the Kalahari. In Cooperative breeding in vertebrates p. 294. (10.1017/CBO9781107338357)

[28] Manser MB , Jansen D , Graw B , Hollen LI , Bousquet CAH , Furrer RD , le Rouex A . 2014 Vocal complexity in Meerkats and other Mongoose species. Adv. Study Behav **46** , 281–310. (10.1016/B978-0-12-800286-5.00006-7)

[29] Reber SA , Townsend SW , Manser MB . 2013 Social monitoring via close calls in meerkats. Proc. R. Soc. B **280** , 20131013. (10.1098/rspb.2013.1013) PMC3712447 23825208

[30] Townsend SW , Zöttl M , Manser MB . 2011 All clear? Meerkats attend to contextual information in close calls to coordinate vigilance. Behav. Ecol. Sociobiol. **65** , 1927–1934. (10.1007/s00265-011-1202-6)

[31] Townsend SW , Hollén LI , Manser MB . 2010 Meerkat close calls encode group-specific signatures, but receivers fail to discriminate. Anim. Behav. **80** , 133–138. (10.1016/j.anbehav.2010.04.010)

[32] Demartsev V , Strandburg-Peshkin A , Ruffner M , Manser M . 2018 Vocal turn-taking in meerkat group calling sessions. Curr. Biol. **28** , 3661–3666. (10.1016/j.cub.2018.09.065) 30416063

[33] Rauber R , Manser MB . 2017 Discrete call types referring to predation risk enhance the efficiency of the meerkat sentinel system. Sci. Rep. **7** , 44436. (10.1038/srep44436) 28303964 PMC5358691

[34] Manser M . 1998 The evolution of auditory communication in Suricates Suricata suricatta. PhD thesis, University of Cambridge, UK.

[35] Wyman MT , Rivers PR , Muller C , Toni P , Manser MB . 2017 Adult meerkats modify close call rate in the presence of pups. Curr. Zool. **63** , 349–355. (10.1093/cz/zox029) 29491994 PMC5804182

[36] Engesser S , Manser MB . 2022 Collective close calling mediates group cohesion in foraging meerkats via spatially determined differences in call rates. Anim. Behav. **185** , 73–82. (10.1016/j.anbehav.2021.12.014)

[37] Gall GEC , Manser MB . 2017 Group cohesion in foraging meerkats: follow the moving 'vocal hot spot.' R. Soc. Open Sci. **4** , 170004. (10.1098/rsos.170004) 28484628 PMC5414265

[38] Rauber R , Manser MB . 2018 Experience of the signaller explains the use of social versus personal information in the context of sentinel behaviour in meerkats. Sci. Rep. **8** , 11506. (10.1038/s41598-018-29678-y) 30139953 PMC6107524

[39] Kenward RE . 2000 A manual for wildlife radio tagging. Cambridge, MA: Academic Press.

[40] Averly B , Sridhar VH , Demartsev V , Gall G , Manser M , Strandburg-Peshkin A . 2022 Disentangling influence over group speed and direction reveals multiple patterns of influence in moving meerkat groups. Sci. Rep. **12** , 13844. (10.1038/s41598-022-17259-z) 35974046 PMC9381760

[41] Gwet KL . 2008 Computing inter-rater reliability and its variance in the presence of high agreement. Br. J. Math. Stat. Psychol. **61** , 29–48. (10.1348/000711006X126600) 18482474

[42] R Development Core Team . 2020 R: a language and environment for statistical computing. Vienna, Austria: R Foundation for Statistical Computing.

[43] Gamer M , Lemon J , Fellows I , Singh P . 2019 Various Coefficients of Interrater Reliability and Agreement. See https://CRAN.R-project.org/package=irr.

[44] Snyder JP . 1987 Map projections—A working manual. In US geological survey professional paper 1395, pp. 38–75. Washington DC: US Government Printing Office.

[45] Thomas M , Jensen FH , Averly B , Demartsev V , Manser MB , Sainburg T , Roch MA , Strandburg-Peshkin A . 2022 A practical guide for generating unsupervised, spectrogram-based latent space representations of animal vocalizations. J. Anim. Ecol. **91** , 1567–1581. (10.1111/1365-2656.13754) 35657634

[46] Bousquet CAH , Sumpter DJT , Manser MB . 2011 Moving calls: a vocal mechanism underlying quorum decisions in cohesive groups. Proc. R. Soc. B **278** , 1482–1488. (10.1098/rspb.2010.1739) PMC3081741 21047853

[47] Karp D , Manser MB , Wiley EM , Townsend SW . 2014 Nonlinearities in meerkat alarm calls prevent receivers from habituating. Ethology **120** , 189–196. (10.1111/eth.12195)

[48] Gall GEC , Strandburg-Peshkin A , Clutton-Brock T , Manser MB . 2017 As dusk falls: collective decisions about the return to sleeping sites in meerkats. Anim. Behav **132** , 91–99. (10.1016/j.anbehav.2017.08.001)

[49] Bosshard AB , Leroux M , Lester NA , Bickel B , Stoll S , Townsend SW . 2022 From collocations to call-ocations: using linguistic methods to quantify animal call combinations. Behav. Ecol. Sociobiol. **76** , 122. (10.1007/s00265-022-03224-3) 36034316 PMC9395491

[50] Gries ST , Stefanowitsch A . 2004 Extending collostructional analysis: a corpus-based perspective on alternations. Int. J. Corpus Linguistics **9** , 97–129. (10.1075/ijcl.9.1.06gri)

[51] Leroux M , Chandia B , Bosshard AB , Zuberbühler K , Townsend SW . 2022 Call combinations in chimpanzees: a social tool? Behav. Ecol. **33** , 1036–1043. (10.1093/beheco/arac074)

[52] Gries ST .2022 Coll.analysis 4.0. A script for R to compute perform collostructional analyses. https://www.stgries.info/teaching/groningen/index.html.

[53] Townsend SW , Allen C , Manser MB . 2012 A simple test of vocal individual recognition in wild meerkats. Biol. Lett. **8** , 179–182. (10.1098/rsbl.2011.0844) 21992821 PMC3297395

[54] Ramos-Fernández G . 2005 Vocal communication in a fission-fusion society: do spider monkeys stay in touch with close associates? Int. J. Primatol. **26** , 1077–1092. (10.1007/s10764-005-6459-z)

[55] Radford AN , Ridley AR . 2008 Close calling regulates spacing between foraging competitors in the group-living pied babbler. Anim. Behav. **75** , 519–527. (10.1016/j.anbehav.2007.05.016)

[56] Radford AN . 2004 Vocal mediation of foraging competition in the cooperatively breeding green woodhoopoe (Phoeniculus purpureus). Behav. Ecol. Sociobiol. **56** , 279–285. (10.1007/s00265-004-0785-6)

[57] Doolan SP , Macdonald DW . 1996 Diet and foraging behaviour of group‐living meerkats, Suricata suricatta, in the southern Kalahari. J. Zool. **239** , 697–716. (10.1111/j.1469-7998.1996.tb05472.x)

[58] Hirsch BT . 2002 Social monitoring and vigilance behavior in brown capuchin monkeys (Cebus apella). Behav. Ecol. Sociobiol. **52** , 458–464. (10.1007/s00265-002-0536-5)

[59] Kondo N , Watanabe S . 2009 Contact calls: information and social function. Jpn. Psychol. Res **51** , 197–208. (10.1111/j.1468-5884.2009.00399.x)

[60] Rendall D , Cheney DL , Seyfarth RM . 2000 Proximate factors mediating 'contact' calls in adult female baboons (Papio cynocephalus ursinus) and their infants. J. Comp. Psychol. **114** , 36–46. (10.1037/0735-7036.114.1.36) 10739310

[61] Caine NG , Stevens C . 1990 Evidence for a “monitoring call” in red-bellied tamarins. Am. J. Primatol. **22** , 251–262. (10.1002/ajp.1350220405) 31952418

[62] McDonald MV , Greenberg R . 1991 Nest departure calls in female songbirds. The Condor **93** , 365–373. (10.2307/1368952)

[63] Sueur C . 2011 Group decision-making in chacma baboons: leadership, order and communication during movement. BMC Ecol. **11** , 26. (10.1186/1472-6785-11-26) 22014356 PMC3224468

[64] Winger BM , Weeks BC , Farnsworth A , Jones AW , Hennen M , Willard DE . 2019 Nocturnal flight-calling behaviour predicts vulnerability to artificial light in migratory birds. Proc. R. Soc. B **286** , 20190364. (10.1098/rspb.2019.0364) PMC6501673 30940055

[65] Bramble DM . 2015 Axial-appendicular dynamics and the integration of breathing and gait in mammal. Am. Zool. **29** , 171–186. (10.1093/icb/29.1.171)

[66] Gustison ML , Borjon JI , Takahashi DY , Ghazanfar AA . 2019 Vocal and locomotor coordination develops in association with the autonomic nervous system. Elife **8** , e41853. (10.7554/eLife.41853) 31310236 PMC6684270

[67] Laplagne DA , Elías Costa M . 2016 Rats synchronize locomotion with ultrasonic vocalizations at the subsecond time scale. Front. Behav. Neurosci. **10** , 184. (10.3389/fnbeh.2016.00184) 27746726 PMC5040720

[68] Demartsev V , Strandburg-Peshkin A . 2024 Mapping vocal interactions in space and time differentiates signal broadcast versus signal exchange in meerkat groups. Mendeley data, V1 (10.17632/f6y7pcvz9p.1) PMC11391280 38768207

[69] Demartsev V , Averly B , Johnson-Ulrich L , Sridhar V , Leonardos L , Thomas M , Manser MB , Strandburg-Peshkin A . 2024 Supplementary Material from: Mapping vocal interactions in space and time differentiates signal broadcast vs signal exchange in meerkat groups. Figshare (10.6084/m9.figshare.c.7198675) PMC11391280 38768207

